# Otitis Media with Effusion in Children and the Impact of Risk Factors on Serum Cytokine Levels

**Published:** 2017-03

**Authors:** Saba Arshi, Fatemeh Dehghani Firouzabadi, Babak Ghalehbaghi, Ali Dehghani Firouzabadi, Farhad Jalali, Mehdi Shekarabi, Reza Sirous, Mohammad Dehghani Firouzabadi

**Affiliations:** 1 *Department of Allergy and Clinical Immunology, Iran University of Medical Sciences, Tehran, Iran.*; 2 *ENT and Head and Neck Research Center and Department* *, Hazrat Rasoul Akram Hospital, Iran University of Medical Sciences,Tehran,Iran.*; 3 *Department ofHealth, Epidemiologist, Mississippi State, United States.*; 4 *Department of Radiology, University of Mississippi Medical Center, Mississippi State, United States.*

**Keywords:** Interleukins, Inflammatory cytokines, Otitis media with effusion, Pediatrics

## Abstract

**Introduction:**

To evaluate the role of allergic-type and infectious-type cytokines in children with chronic otitis media with effusion (OME)

**Materials and Methods:**

We investigated serum levels of interleukins (IL)-4, IL-5, and IL-13, along with interferon-gamma (IFN-γ) and tumor necrosis factor-alpha (TNF-α), by enzyme-linked immunosorbent assay (ELISA) in 35 children with OME and 28 healthy controls.

**Results:**

Children with OME had significantly higher levels of IL-5 in comparison with the control group, ranging from 1 pg/ml in cases to 0.04 pg/ml in controls (P=0.009). However, after adjusting for confounding variables, there was no significant difference in serum levels of IL-13, IL-4, IFN-γ, or TNF-α between the two groups (P=0.287, P=0.627, P=0.793, and P=0.217, respectively)

**Conclusions:**

The findings of this study suggest that in comparison with the control group, serum IL-5 levels were elevated in OME cases.

## Introduction

Otitis media with effusion (OME) is defined as the presence of fluid in the middle ear, without signs or symptoms of acute ear infection, involving fever and acute pain (1,2). Causes of OME include recurrent infections, inflammatory conditions, malformations, and tube dysfunctions (3–5). The treatment of choice in patients with persistent OME is tympanostomy tube replacement, which is the most common pediatric surgical procedure requiring anesthesia in the United States (US) (6,7). Epidemiologic reports of OME in children have been published over the past decade and have evaluated the prevalence of OME in this population. For example, in young Australian aboriginal children, OME was reported to affect 41% of infants under the age of 18 months (8).

 According to another estimate, approximately 2.2 million diagnosed episodes of OME occur annually in the US, yielding a combined direct and indirect annual cost estimate of $4.0 billion (2). Moreover, OME may cause long-term sequelae such as delayed language development and learning difficulties. Indeed, young children with OME show reduced articulation, while older children often show attention deficit in school (5,9-11).

Susceptibility to OME is multifactorial, and includes environmental, microbial, and host-related factors such as allergic predisposition and immune responses. Many of these factors have been discussed in previous studies (12,13). However, our knowledge of immune responses to allergic and non-allergic factors is not complete. Recently the “hygiene hypothesis” proposed that decreased infectious and non-allergic immune responses via T-helper-1 (TH-1) in early childhood can lead to higher allergic responses via T-helper-2 (TH-2) cells (14, 15). It is not clear if children with persistent OME have higher systemic levels of allergic-type TH-2 cell mediators such as interleukin (IL)-4, IL-5, and IL-13 than control subjects (16,17).

The primary goal of this study was to investigate allergic-type TH-2 cell mediators such as IL-4, IL-5, and IL-13 and infectious-type cytokines such as interferon-gamma (IFN-γ) and tumor necrosis factor-alpha (TNF-α) in children with persistent OME. The secondary goal was to investigate any potential correlation between the level of cytokines and risk factors associated with OME.

## Materials and Methods

*Patient enrollment and study design: *From January 2014 to August 2014, 63 subjects (cases and controls) were recruited at the Pediatric Clinic of Hazrat-e-Rasool Akram Hospital (a teaching hospital affiliated with Iran University of Medical Sciences, Tehran, Iran). Inclusion criteria for enrolling eligible cases were a history of chronic or recurrent otitis media (OM) treated with tubes. Cases were excluded if they had a history of Down’s syndrome, immune deficiency, cleft palate or other craniofacial anomaly, or any other condition predisposing to OM. Twenty-eight healthy children without OME were chosen as a control group. Subjects in the control group had two or fewer OM episodes per year and did not have a history of tubes. Exclusion criteria for the control group were the same as for the case group. Written informed consent was obtained from the parents of all study participants. The study protocol was approved by the Iran University of Medical Sciences (IUMS) Ethics Committee. A short medical history and risk factors of OME were collected for all children. Venous blood samples were taken for cytokine assays. Blood samples were frozen at −70 °C and enzyme-linked immunosorbent assay (ELISA) for serum IL-4, IL-5, IL-13, IFN-γ, and TNF-α were performed for both cases and controls.

*Statistical analysis:*

 Baseline characteristics of the study participants were compared using an independent t-test or chi-square test, where appropriate. Continuous variables with skewed distribution were analyzed using the Mann Whitney test. Analysis of covariance (ANCOVA) models were used with the four factors that were significant by univariate analyses. SPSS version 22.0 (IBM Corporation, New York, US) was used for statistical analyses. P<0.05 was considered statistically significant.

## Results

Thirty-five cases and 28 controls were matched for age, gender and body mass index (BMI) (P=0.954, P=0.628, P=0.972, respectively). Ages ranged from 8 months to 12 years and 8 months in the case group and from 2 years and 4 months to 12 years and 8 months in the control group. Sixty-five percent of cases and 71% of controls were male. No differences were found between the two groups in terms of exposure to tobacco smoke, level of parental education, history of asthma, allergic rhinitis, food allergies, urticarial or atopic eczema, type of delivery, family size, breastfeeding, or history of ventilation tube (VT) insertion in siblings (Table 1). However, daycare attendance, history of adenotonsillectomy and VT insertion were more frequent among cases (P=0.01, P=0.012, P=0.03, respectively), while use of a pacifier was less common among cases (P=0.01; Table.1).

**Table 1:** Baseline characteristics of cases and controls

	**Cases (n=35)**	**Controls (28)**	**P values**
Gender (♂)	23 (65.7%)	20 (71.4%)	0.628*
Age (year)	6.6 ±2.3	6.5 ±2.6	0.954**
Weight(kg)	24.65 ±9.8	24.73 ±8.6	0.972**
Height (cm)	118 ±17	120 ±17.7	0.629**
BMI (kg/m2)	24.65 ±9.8	24.73 ±8.6	0.972**
Hx Cigarette smoke exposure (father)	10 (28.6%)	10(35%)	0.413*
Hx Cigarette smoke exposure (mother)	0(0%)	2(7.1%)	0.111*
Asthma	3(8.7%)	3(10.7%)	0.775*
Food allergies	3(8.6%)	0(0%)	0.112*
Type of delivery (caesarian)	19(54%)	14(50%)	0.735*
Family size	3.91 ±0.85	3.71 ±0.93	0.231**
Allergic rhinitis(yes)	12(34.3%)	4(14.3%)	0.643*
VT sibling(yes)	1(2.9%)	0(0%)	0.367*
Day care(yes)	19(54%)	6(22%)	0.021*
Breast milk(yes)	33(94%)	25(88%)	0.942*
Formula (yes)	5(14.4%)	3(10.2%)	0.308*
Atopic eczema(yes)	1(2.9%)	0(0%)	0.367*
Urticaria (yes)	2(5.7%)	3(10.7%)	0.249*
History of adenotonsilectomy	7(20%)	0(0%)	0.012*
Pacifier usage	2(5.7%)	8(28.6%)	0.014*
VT insertion in patients	2(5.7%)	0(0%)	0.045*
Education level(less than high school)	17(48%)	13(46%)	0.43 *

*: Chi squared test

**: T test

Univariate analyses showed that serum levels of IL-5 were significantly higher in cases than controls (1 versus 0.04 pg/ml; P=0.009). However, there were no significant difference in serum levels of IL-13, IL-4, IFN-γ, or TNF-α between the two groups (P=0.287, P=0.627, P=0.793, P=0.217, respectively; Table.2).

After adjustment for confounding variables such as history of daycare attendance, adenotonsil- lectomy, previous VT insertion in patients, and use of a pacifier in the multivariate linear regression model, the results showed that there was no correlation between these predictors and increased serum levels of IL-5 (P=0.445,P=0.602,P=0.188,P=0.627,respectively); therefore levels of this cytokine were not confounded by these variables. Therefore, based on these results, the serum level of IL5 was significantly higher among cases and was not affected by other variables (P=0.009; Table.2).

**Table 2:** comparison of serum cytokines in cases and controls

	**Cases (35)**	**Controls (28)**	**P values**
IL-13	3.47 ±5.24	1.57 ±1.75	0.287***
IL-4	1.72 ±4.60	1.20 ±2.68	0.627***
IL-5	1 ±2.26	0.04 ± 0.21	0.009***
TNF-α	1.46 ±3.70	0.74 ±1.97	0.217***
INF-gamma	2.56 ±4.61	1.92 ± 1.60	0.793***

INF-γ=interferon-gamma, TNF-α=tumor necrosis factor- alpha, IL-4=interleukin-4, IL-5 =interleukin-5, IL-13=interleukin-13

***: Mann-Whitney U test

## Discussion

OME is one of the most common diseases in pediatric practice and causes a detrimental effect on healthcare throughout the world, reducing quality of life in affected children (5, 9–11,18). Among many factors which cause OME, it seems that allergic processes mediated by cytokines play a role (12,13). Many previous studies have demonstrated a predominance of TH-2 mediators in the middle ear effusions (MEEs) of atopic children (8,19–27). A 2015 study by Zielnik-Jurkiewicz et al. reported that TNF-α was found in the effusion collections of all children with chronic OME (20), while Wright et al. demonstrated an elevated level of IL-5 in the middle ear mucosa of atopic children with persistent OME (21). In a review article, Smironova et al. showed that the presence of cytokines, including IL-4 and IL-5, identified in the otitis media were responsible for chronic inflammation of the middle ear and chronic OME (26). One of the few studies evaluating systemic cytokines in this condition was reported by Johnston et al. and showed a systemic response consistent with a TH-2-type response. This study compared cytokine levels (IL-4, IL-5, TNF-α, and INF-γ) in children with and without chronic or recurrent OM. Although on univariate analysis, children with OME had increased levels of serum IL-5 and INF-γ compared with children without OME, serum levels of INF-γ in children with OME were not significantly higher than controls after multivariate analyses (28). In current study, we found that serum levels of IL-5 (one of the most important TH-2-type cytokines) were significantly higher in children with OME in comparison with those without OME (P=0.009), but that there was no significant difference in serum levels of IL13, IL-4, IFN-γ, or TNF-α between children with and without OME. This is similar to findings of previous studies.

Our study confirms a TH-2-type immune response role in chronic OME, as demonstrated by higher levels of systemic IL-5 in children with OME versus controls. Previous studies have also demonstrated a similar finding (20–24). It is not clear whether the increased level of systemic IL-5 is the etiologic mechanism of OME or the result of OME itself.

Similar to previous studies, this study showed no significant association between gender and prevalence of OME (29,30). Previous studies have demonstrated that prevalence of OME is associated with age (30–35). Humaid et al. indicated that age <8 years is one of the most important risk factors of OME (31), while Zielhuis et al. reported that there are two peaks for prevalence of OME; one around 2 years and the other around 5 years of age, and that OME prevalence generally declines after the age of 5 years (36). However, in our study, age was not shown to be a significant risk factor.

The most studied risk factor for OME is exposure to tobacco smoke at home. Similar to our study, Humaid et al. reported no significant correlation between OME and exposure to tobacco smoke (31), while in a cohort of school-aged children, Xenellis et al. showed that OME was not associated with exposure to tobacco smoke (30). However, other studies have shown a statistically significant correlation between exposure to second-hand smoke (SHS) and development of OME (29,37,38). The reason for this may be inhibition of dendritic cell-mediated priming of T-cells by tobacco-smoke components (39). Another study showed that mice that had been exposed to tobacco smoke had increased levels of IL-2, IL-5, and granulocyte-macrophage colony-stimulating factor (GMCSF), alongside a decreased level of INF-γ in their bronchoalveolar lavage (40). Feleszko et al. demonstrated that IL-13 levels were greatly increased in children with tobacco smoke exposure compared with those seen in unexposed children (41). Karen et al. demonstrated that children with SHS exposure have a significantly lower mean concentration of INF-gamma, while children with severe exposure had a significantly lower mean concentration of IL-4 than those with no SHS exposure (42).

Recent studies have demonstrated the role of atopy and/or allergy in the pathogenesis of OME (29,32,43,44). Researchers have shown that among allergic diseases, the prevalence of allergic rhinitis and adenoid hypertrophy were significantly higher in children with OME (25). However, similar to our study, some studies have suggested that there is no difference in the prevalence of allergic diseases such as rhinoconjunctivitis, asthma, or eczema between children with and without OME (33,45,46).

Similar to our study, there was no difference in family size between children with and without OME (32). However, in contrast, the study conducted by Humaid et al. found that the number of family members in the household is a predictor of OME (31). Our study is similar to previous studies that reported no significant correlation between prevalence of OME and being breastfed during the first 2 years of life, either exclusively or in combination with formula (29–31). On the other hand, another study reported that a history of no breastfeeding is associated with a significantly higher prevalence of OME (32). Our results are similar to those reported by Caylan et al. suggesting that daycare attendance is a significant risk factor for OME (35), although another study found no significant relationship between OME and daycare attendance (31).

There was no significant difference between type of delivery, history of VT insertion in siblings, history of adenotonsillectomy, or history of VT insertion between the case and the control groups. Similar to a study by Sophia et al., the educational level of the mother was not an influential factor for OME (29). Although previous studies have shown that pacifier use is associated with an increase in the incidence of otitis media (47,48), we found pacifier use not to be a significant risk factor for increased prevalence of OME. It can be concluded from our study that gender, age, BMI, daycare attendance, family size, asthma, food allergies, allergic rhinitis, atopic eczema, urticaria, type of delivery, VT insertion in siblings, parental educational level, type and duration of breastfeeding, or exposure to passive smoking were not risk factors for OME. However, history of daycare attendance, adenotonsillectomy, VT insertion in patients, and pacifier usage were correlated with high OME rates.

In conclusion, the results of the present study are consistent with previous studies suggesting that pro-inflammatory cytokine levels do not differ significantly between children with exposure to tobacco smoke, asthmatic children, and children with food allergies and those who do not have these predisposing factors. In this study, these risk factors were all included in the model to account for their contribution to cytokine levels. However, there was a significant difference in the serum levels of IL-5 between cases and controls. Thus, the present study demonstrates that serum levels of cytokines are elevated in OME, and this result is similar to previous studies showing elevated TH-2-type cytokine levels in MEEs and serum. This finding is relevant clinically because localized treatment of recurrent OME is not sufficient and the condition is associated with a systemic response.
